# The performance of artificial intelligence-driven technologies in diagnosing mental disorders: an umbrella review

**DOI:** 10.1038/s41746-022-00631-8

**Published:** 2022-07-07

**Authors:** Alaa Abd-alrazaq, Dari Alhuwail, Jens Schneider, Carla T. Toro, Arfan Ahmed, Mahmood Alzubaidi, Mohannad Alajlani, Mowafa Househ

**Affiliations:** 1 AI Center for Precision Health, Weill Cornell Medicine-Qatar, Doha, Qatar; 2 Information Science Department, Kuwait University, Alshadadiya, Kuwait; 3 Health Informatics Unit, Dasman Diabetes Institute, Kuwait city, Kuwait; 4 Division of Information and Computing Technology, College of Science and Engineering, Hamad Bin Khalifa University, Qatar Foundation, Doha, Qatar; 5 Institute of Digital Healthcare, University of Warwick, Warwick, UK

**Keywords:** Health care, Medical research, Diseases, Psychiatric disorders

## Abstract

Artificial intelligence (AI) has been successfully exploited in diagnosing many mental disorders. Numerous systematic reviews summarize the evidence on the accuracy of AI models in diagnosing different mental disorders. This umbrella review aims to synthesize results of previous systematic reviews on the performance of AI models in diagnosing mental disorders. To identify relevant systematic reviews, we searched 11 electronic databases, checked the reference list of the included reviews, and checked the reviews that cited the included reviews. Two reviewers independently selected the relevant reviews, extracted the data from them, and appraised their quality. We synthesized the extracted data using the narrative approach. We included 15 systematic reviews of 852 citations identified. The included reviews assessed the performance of AI models in diagnosing Alzheimer’s disease (*n* = 7), mild cognitive impairment (*n* = 6), schizophrenia (*n* = 3), bipolar disease (*n* = 2), autism spectrum disorder (*n* = 1), obsessive-compulsive disorder (*n* = 1), post-traumatic stress disorder (*n* = 1), and psychotic disorders (*n* = 1). The performance of the AI models in diagnosing these mental disorders ranged between 21% and 100%. AI technologies offer great promise in diagnosing mental health disorders. The reported performance metrics paint a vivid picture of a bright future for AI in this field. Healthcare professionals in the field should cautiously and consciously begin to explore the opportunities of AI-based tools for their daily routine. It would also be encouraging to see a greater number of meta-analyses and further systematic reviews on performance of AI models in diagnosing other common mental disorders such as depression and anxiety.

## Introduction

Mental disorders affect a person’s psychological, social, behavioral, and emotional wellbeing^1^. The impact of mental disorders is not exclusive to the mind; one’s mental health state affects physical wellbeing and vice-versa^2^. Globally, mental disorders account for 7% of all total disability-adjusted life years (DALYs) and affect more than 1 billion people, especially those living in high and upper-middle-income nations^3^. This burden is further exacerbated by the fact that up to 50% and 90% of people with mental disorders receive no treatment in high-income countries and low resource settings, respectively^4^.

Diagnosing mental disorders is complicated by heterogeneity in clinical presentation, symptomatology, and fluctuations in the course of illness, further compounded by gaps in our understanding of etiological mechanisms. Current practices to diagnose mental disorders rely on frameworks outlined in the Diagnostic and Statistical Manual of Mental Disorders (DSM-5) and the International Classification of Diseases (ICD-11) manual. Diagnosis is based entirely on subjective accounts from patients on the one hand and observations and interpretations made by clinicians on the other; objective measures are still not available^5^. Furthermore, diagnosing mental disorders can be time- and resource-intensive via administering diagnostic tools, conducting interviews with relatives or caregivers, and taking health histories.

Digital health tools and technologies offer great opportunities to support and augment diagnostic and interventional aspects of psychiatric care^6^. A leading and popular form of such digital technologies is artificial intelligence (AI), which enables machines to learn complex, latent rules and provide actionable conclusions through understanding queries and sifting through and connecting mountains of data points^7^. Advances in the use of AI for diagnostic and therapeutic mental health interventions are on the rise with multiple examples including social bots to support dementia care, sexual disorders, and even virtual psychotherapists^8–11^. AI has great potential to reshape our understanding of mental disorders and how to diagnose them. Leveraging AI to study and make sense of complex patterns and interactions between one’s genes, brain, behaviors, and experiences present an unprecedented opportunity to improve early mental illness detection and personalize treatment options^5^.

There have been a wealth of studies examining the accuracy of AI models in diagnosing mental disorders such as Alzheimer’s Disease (AD)^12^, schizophrenia (SCZ)^13^, bipolar disorders (BD)^14^, posttraumatic stress disorders (PTSD)^15^, and obsessive-compulsive disorder (OCD)^16^. Numerous systematic reviews summarize the evidence resulting from these studies. Although conducting an umbrella review (i.e., a review of systematic reviews) is important to draw more accurate and comprehensive conclusions on a particular topic, to our knowledge, no previous umbrella reviews were published to summarize the evidence about diagnostic performance of AI models for mental disorders. This umbrella review aims to synthesize the previously published evidence on the performance of AI models in diagnosing mental disorders.

## Results

### Search Results

**Fig. 1:**
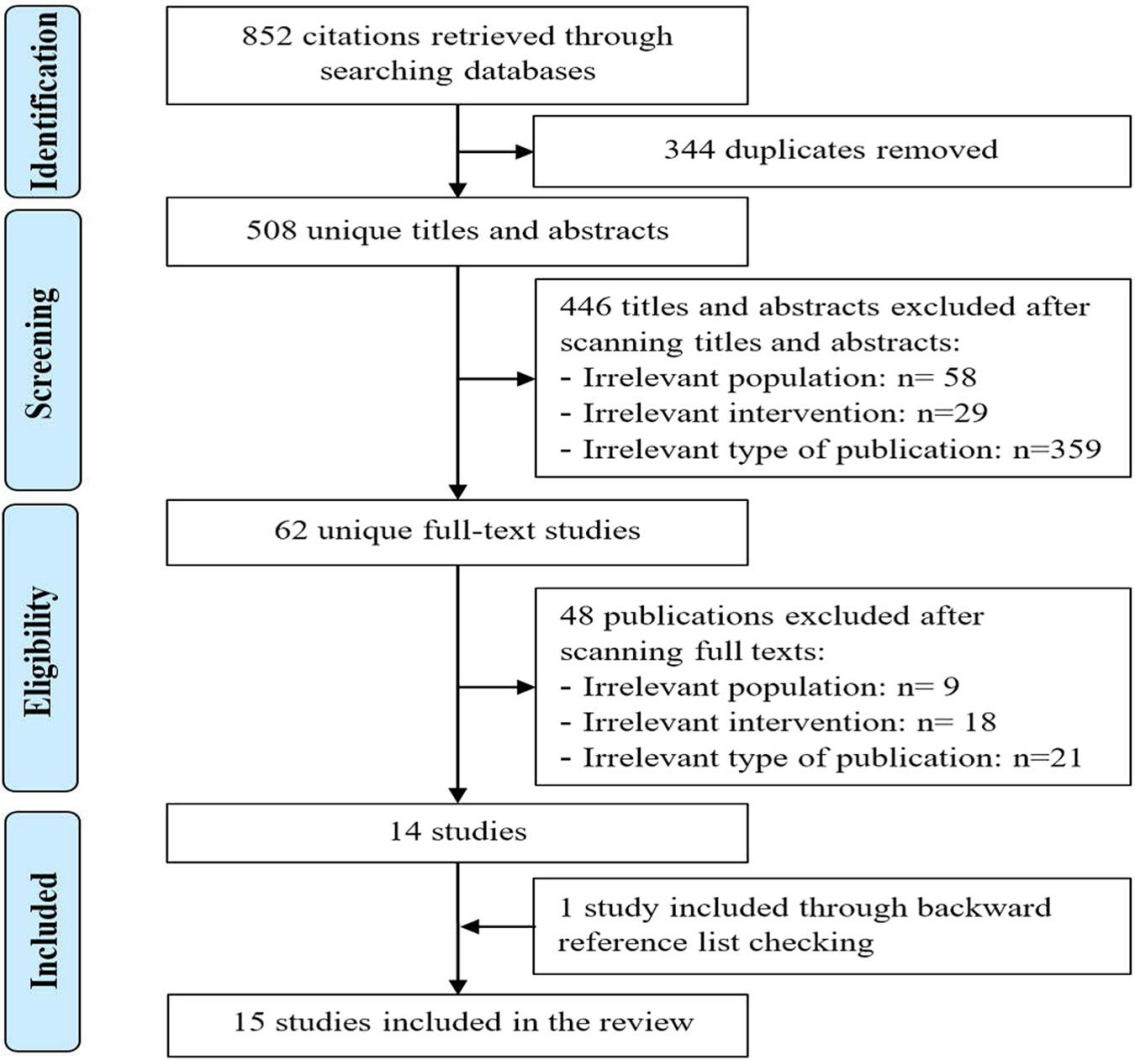
Flow chart of the study selection process: 852 citations were retrieved from searching the databases. Of these, 344 duplicates were removed. Screening titles and abstracts of the remaining citations led to excluding 446 citations. By reading the full text of the remaining 62 publications, we excluded 48 publications. An additional systematic review was identified by checking the list of the included reviews. In total, 15 systematic reviews were included in the current.

As presented in Fig. 1, we identified a total of 852 citations from searching the bibliographic databases. The software EndNote identified and removed 344 duplicates of the retrieved citations. Screening titles and abstracts of the remaining 508 citations led to excluding 446 citations. By reading the full text of the remaining 62 publications, we excluded 48 publications. An additional systematic review was identified through checking the list of the included reviews. In total, 15 systematic reviews were included in the current review^17–31^.

### Characteristics of included reviews

Interestingly, the included reviews were published between 2017 and 2020, and more than half of them (*n* = 8) were published in 2020 (Table 1). The included reviews were conducted in 7 different countries, but more than half of them were conducted in Italy (*n* = 5) and the United Kingdom (*n* = 4). All included reviews were articles in peer-reviewed journals. Only four reviews had a registered protocol. All studies except one stated that they followed Preferred Reporting Items for Systematic Reviews and Meta-Analyses (PRISMA) guidelines.

**Table 1:** Meta-data of the included reviews.

Study	Year	Country	Publication type	Registered protocol	Followed guidelines
Pellegrini^17^	2018	UK	Journal article	Yes	PRISMA^a^
Billeci^18^	2020	Italy	Journal article	No	PRISMA^a^
Sarica^19^	2017	Italy	Journal article	No	PRISMA^a^
Ebrahimighahnavieh^20^	2020	Australia	Journal article	No	No
Petti^21^	2020	UK	Journal article	No	PRISMA^a^
Battista^22^	2020	Italy	Journal article	No	PRISMA^a^
Law^23^	2020	UK	Journal article	No	PRISMA^a^
de Filippis^24^	2019	Italy	Journal article	No	PRISMA^a^
Steardo^25^	2020	Italy	Journal article	No	PRISMA^a^
Bracher-Smith^26^	2020	UK	Journal article	Yes	PRISMA^a^
Librenza-Garcia^27^	2017	Brazil	Journal article	No	PRISMA^a^
Moon^28^	2019	South Korea	Journal article	Yes	PRISMA^a^
Ramos-Lima^29^	2019	Brazil	Journal article	Yes	PRISMA^a^
Bruin^30^	2019	Netherlands	Journal article	No	PRISMA^a^
Sanfelici^31^	2020	Germany	Journal article	No	PRISMA^a^

^a^*PRISMA* Preferred Reporting Items for Systematic Reviews and Meta-Analyses, *UK* United Kingdom.

With regards to the eligibility criteria, the included studies focused on diagnosing 10 mental disorders, namely: Alzheimer’s disease (AD) (*n* = 7), mild cognitive impairment (MCI) (*n* = 6), and Schizophrenia (SCZ) (*n* = 3) (Table 2). While seven reviews focused on any AI approach, another seven reviews focused merely on supervised machine learning (SML), and one review focused on deep learning (DL). SML uses labeled datasets to train algorithms in order to predict or label new, unforeseen examples, SML is used for classification and regression purposes. UML analyzes unlabeled data to discover hidden features, patterns, and relationships in data. Clustering, association, and dimensionality reduction are three major applications of unsupervised learning models. It is worth mentioning that most deep learning applications are based on supervised learning. More than half of the reviews (*n* = 8) focused on neuroimaging data for diagnosing mental disorders. While seven reviews restricted the search to studies in the English language, there was no language restriction imposed in six studies. Eight studies applied time restrictions to the search while the remaining studies did not.

**Table 2:** Eligibility criteria of the included reviews.

Study	Target disorder	AI approach	Type of data	Language restrictions	Time limit
Pellegrini^17^	AD & MCI	UML, SML, DL	Neuroimaging data	No restriction	January 1, 2006–September 30, 2016
Billeci^18^	AD & MCI	SML	Neuroimaging data	NR	January 1, 2010–2019
Sarica^19^	AD & MCI	SML	Neuroimaging data	English	January 1, 2007–May 1, 2017
Ebrahimighahnavieh^20^	AD & MCI	DL	Neuroimaging data	English	No restriction
Petti^21^	AD & MCI	UML, SML, DL	Neuropsychological tests	English	January 1, 2013–August 8, 2019
Battista^22^	AD & MCI	SML	Neuropsychological tests	English	January 1, 2010–July 15, 2018
Law^23^	AD & DLB	SML	EEG measures	English	No restriction
de Filippis^24^	SCZ	UML, SML, DL	Neuroimaging data	No restriction	No restriction
Steardo^25^	SCZ	SML	Neuroimaging data	No restriction	No restriction
Bracher-Smith^26^	SCZ, BD, ASD, AN	UML, SML, DL	Genetic data	English	No restriction
Librenza-Garcia^27^	BD	UML, SML, DL	No restriction	No restriction	January 1, 1960–January 1, 2017
Moon^28^	ASD	UML, SML, DL	Neuroimaging data	No restriction	No restriction
Ramos-Lima^29^	PTSD	UML, SML, DL	No restriction	No restriction	January 1, 1960–May 1, 2019
Bruin^30^	OCD	SML	Neuroimaging data	NR	No restriction
Sanfelici^31^	Psychotic disorders	SML	No restriction	English	No restriction

*AD* Alzheimer’s disease, *AI* Artificial intelligence, *AN* Anorexia nervosa, *ASD* Autism spectrum disorder, *BD* Bipolar disease, *DL* Deep learning, *DLP* Dementia with Lewy bodies, *EEG* Electroencephalography, *MCI* Mild cognitive impairment, *NR* Not reported, *OCD* Obsessive-compulsive disorder, *PTSD* Post-traumatic stress disorder, *SCZ* Schizophrenia, *SML* Supervised machine learning, *UML* Unsupervised machine learning.

Varied numbers of electronic databases were searched in the included reviews. The most common databases used in the included reviews are MEDLINE (*n* = 13), Web of Science (*n* = 7), EMBASE (*n* = 6), PsycINFO (*n* = 5), and Scopus (*n* = 4) (Table 3). Eight studies used either backward reference list checking (*n* = 7) or forward reference list checking (*n* = 1) to identify further studies. Two independent reviewers carried out the study selection process in twelve reviews, performed data extraction in four reviews, and assessed study quality in two reviews. The quality of studies was assessed in nine reviews using six different tools such as a revised tool for Quality Assessment of Diagnostic Accuracy Studies (QUADAS-2) and Jadad rating system. Four reviews synthesized the data using meta-analysis.

**Table 3:** Search sources, study selection, data extraction, quality assessment, and data synthesis in the included reviews.

Study	Databases searched	Reference list checking	Number of reviewers			Quality assessment tool	Meta-analysis
			Study selection	Data extraction	Quality assessment		
Pellegrini^17^	MEDLINE, Elsevier, IEEE Xplore, Science Direct, ACM Digital Library, Web of Science	No	2	NR	NR	QUADAS-2	No
Billeci^18^	MEDLINE	No	2	NR	NA	No	No
Sarica^19^	MEDLINE, Scopus, Web of Science, Google Scholar	No	2	NR	NA	No	No
Ebrahimighahnavieh^20^	IEEE Xplore, ScienceDirect, SpringerLink, ACM Digital Library, Web of Science, Scopus	Forward	1	NR	NR	Tool developed by the authors	No
Petti^21^	MEDLINE, Web of Science, Ovid	No	2	NR	NA	No	No
Battista^22^	NR	Backward	2	NR	NR	QUADAS-2	Yes
Law^23^	MEDLINE, EMBASE, PsycINFO	Backward	2	2	NR	Joanna Brigg Institute	No
de Filippis^24^	MEDLINE, EMBASE, PsycINFO, Cochrane Library	Backward	2	NR	NR	Jadad rating system	No
Steardo^25^	MEDLINE, EMBASE, PsycINFO, Cochrane Library	Backward	2	2	NR	Jadad rating system	No
Bracher-Smith^26^	MEDLINE, PsycINFO, Web of Science, and Scopus	No	2	2	2	PROBAST	No
Librenza-Garcia^27^	MEDLINE, EMBASE, Web of Science	Backward	2	NR	NA	No	Yes
Moon^28^	MEDLINE, EMBASE, CINAHL, PsycINFO, IEEE Xplore	No	2	2	2	QUADAS-2	Yes
Ramos-Lima^29^	MEDLINE, EMBASE, Web of Science	Backward	2	NR	NR	Tool developed by the authors	No
Bruin^30^	MEDLINE	Backward	NR	NR	NA	No	No
Sanfelici^31^	MEDLINE, Scopus	Backward	NR	2	NA	No	Yes

*NA* Not applicable, *NR* Not reported, *PROBAST* Prediction model risk of bias assessment tool, *QUADAS-2* Revised tool for Quality Assessment of Diagnostic Accuracy Studies.

The number of retrieved studies in the included reviews ranged from 52 to 7,991 (Table 4). The number of included studies in the included reviews varied between twelve to 114. The size of data sets used to train and validate models in the included studies ranged between 10 and 7,026 data points. The included studies in the included reviews used different types of data to train and validate models, namely: neuroimaging data (*n* = 13), neuropsychological data (*n* = 6), genetic data (*n* = 4), and Electroencephalography (EEG) measures (*n* = 4). As shown in Table 5, many methods were used in the included studies, and the most common ones were Support Vector Machine (SVM) (*n* = 13), Random Forest (RF) (*n* = 10), Naïve Bayes (NB) (*n* = 7), *k*-Nearest Neighbors (k-NN) (*n* = 5), and Linear Discriminant Analysis (LDA) (*n* = 5). The models in the included reviews were validated using only internal validation methods (*n* = 6) or both internal and external validation methods (*n* = 3).

**Table 4:** Search results and dataset features in the included studies in the included reviews.

Study	# of retrieved studies	# of included studies	Dataset size	Data type
Pellegrini^17^	7991	111	100–902	Neuroimaging data, CSF biomarkers, Demographic data, Genetic data, Biological data
Billeci^18^	52	21	31–330	Neuroimaging data
Sarica^19^	70	12	26–870	Neuroimaging data
Ebrahimighahnavieh^20^	NR	114	43–2,464	Neuroimaging data, Genetic data, Demographical data, Clinical data, CSF biomarkers Neuropsychological test
Petti^21^	2,447	33	10–484	Neuropsychological tests (Speech and language data)
Battista^22^	203	59	22–7,026	Neuropsychological data, Biological data, Neuroimaging data, Demographical data, Clinical data
Law^23^	1,264	43	61–654	EEG measures, Neuroimaging data, CSF biomarkers
de Filippis^24^	2,386	35	34–734	Neuroimaging data
Steardo^25^	660	22	40–737	Neuroimaging data
Bracher-Smith^26^	1,241	13	20–5,554	Genetic data
Librenza-Garcia^27^	757	51	42–4,488	Neuroimaging, Genetic data, EEG measures, Neuropsychological tests, Serum biomarkers
Moon^28^	348	43	20–2,686	Neuroimaging data, EEG measures, Neuropsychological tests, Biochemical data
Ramos-Lima^29^	806	49	25–391	Neuroimaging data, Neuropsychological data, EEG measures, Biological data, Clinical data
Bruin^30^	170	12	20–172	Neuroimaging data
Sanfelici^31^	1,103	44	38–202	Neuroimaging data, Clinical data

*CSF* Cerebrospinal Fluid, *EEG* Electroencephalography.

**Table 5:** Features of models in the included studies in the included reviews.

Study	Classification algorithm type	Type of validation
Pellegrini^17^	Fuzzy, HMM, k-NN, LASSO, LBP, LDA, MIL, NN, PNN, QDA, QDC, RF, RLR, SRC, SVM, ν-MKL	NR
Billeci^18^	AdaBoost, DS, EGB, LDA, LinReg, LogReg, NB, PLSDA, RF, SVM	Internal validation (K-fold cross-validation & Leave One Out cross validation)
Sarica^19^	RF	Internal validation (K-fold cross-validation, Leave One Out cross validation) & External validation
Ebrahimighahnavieh^20^	AE, CNN, DNN, MLP, RNN, DBN, DBM, DPN	Internal validation (K-fold cross-validation, Train-and-test, Leave One Out cross validation)
Petti^21^	DT, LogReg, NB, SVM	NR
Battista^22^	BN, GC, LDA, linReg, LogReg, NB, NN, RF, SVM	Internal validation (K-fold cross-validation, Train-and-test, Nested Cross Validation, Leave One Out cross validation)
Law^23^	RF, SVM	NR
de Filippis^24^	AE, DBN, DNN, ENet, GC, GNet, LASSO, LDA, LogReg, MPA, RDA, RF, Ridge, SRBVS, SVM, TBMFA, ν-MKL	Internal validation (K-fold cross-validation, Leave One Out cross validation)
Steardo^25^	SVM	NR
Bracher-Smith^26^	AdaBoost, BFT, BN, DT, DTNB, EC, GBM, k-NN, LASSO, NB, MDR, NN, RF, Ridge, SVM	Internal validation (K-fold cross-validation, Train-and-test, Leave One Out cross validation, Apparent validation) & External validation
Librenza-Garcia^27^	ANN, BN, CRT, DT, k-NN, LASSO, LR, MFA, MLR, MDL, NB, NN, NSC, RBFN, RF, SVM	NR
Moon^28^	ANN, DNN, DT, Fuzzy, GBM, k-NN, LDA, logReg, MLP, NB, PLSDA, RF, SVM	Internal validation, External validation, and both
Ramos-Lima^29^	SVM, DBN, k-NN, MLP, NB, SMO, TL	NR
Bruin^30^	LogReg, SVM	Internal validation (Leave One Out cross validation & Train-and-test)
Sanfelici^31^	RF, SVM	Internal validation (K-fold cross-validation, Leave One Out cross validation)

*AE* Auto-Encoder, *AN* Anorexia nervosa, *ANN* Artificial Neural Network, *BFT* best-first tree, *BN* Bayesian Network, *CHR* clinical high risk; *CIF* Conditional Inference Forests, *CNN* Convolutional Neural Networks, *CRT* Classification and Regression tree, *DBM* Deep Boltzmann Machine, *DBN* Deep Belief Network, *DNN* Deep Neural Network, *DPN* Deep Polynomial Network, *DS* Decision Stump, *DTNB* Decision Table Naïve Bayes, *EC* Evolutionary Computation, *EGB* Extreme Gradient Boosting, *ENet* Elastic Net, *GBM* Gradient Boosting Machine, *GC* Gaussian Classifier, *GNet* Graph Net, *HMM* Hidden Markov Model, *k-NN* K-Nearest Neighbors, *LASSO* Least Absolute Shrinkage and Selection Operator, *LBP* Local Binary Patterns, *LDA* Linear Discriminant Analysis, *LinReg* Linear Regression, *LogReg* Logistic Regression, *MDL* Minimum Description Length, *MDR* Multifactor Dimensionality Reduction, *MFA* Mixture Factor Analysis, *MIL* Multiple Instance Learning, *MLR* Multivariate Logistic Regressions, *NSC* Nearest Shrunken Centroids, *MLP* Multi-Layer Perceptron, *MPA* Multivariate Pattern Analysis, *NB* Naïve Bayes, *NN* Neural Networks, *NR* Not reported, *OPLS* Orthogonal Projections to Latent Structures, *PLSDA* Partial Least Squares Discrimination Analysis, *PNN* Probabilistic Neural Network, *QDA* Quadratic Discriminant Analysis, *QDC* Quadratic Discriminant Classifier, *RBFN* Radial Basis Function Network, *RDA* Regularized Discriminant Analysis, *RF* Random Forest, *Ridge* Ridge Regression, *RLR* Regularized Logistic Regression, *RNN* Recurrent Neural Network, *SMO* Sequential Minimal Optimization, SRBVS Sparse-Representation-Based Variable Selection, *SRC* Sparse Representation Classification, *SVM* Support Vector Machine, *TBMFA* Translation Based Multimodal Fusion Approach, *TC* trauma-exposed controls, *TL* Transfer Learning, *ν-MKL* Multiple Kernel Learning.

### Results of study quality appraisal

**Fig. 2:**
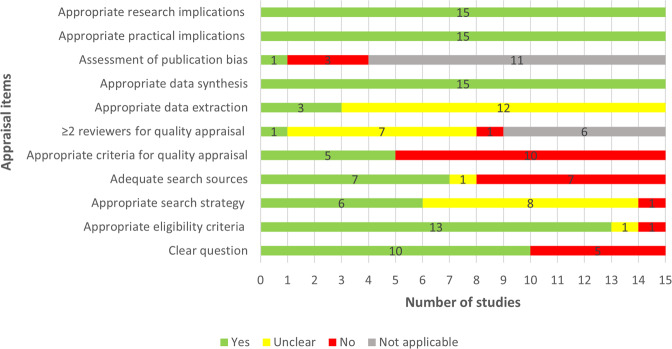
Review authors’ judgments about each appraisal item: The quality of the included reviews was assessed against appraisal items. Yes (green) refers that study meets the item, thereby, it has a good quality in terms of that item. No (red) refers that study did not meet the item, thereby, it has poor quality in terms of that item. Unclear (yellow) refers that we could not appraise the study in terms of the item due to the lack of reported information. Not applicable (gray) refers that the appraisal item is not applicable to the systematic review as it does not include a feature that the item assesses.

Two thirds of the included reviews clearly stated the review question or aim by identifying the AI approach of interest and its aim, the target disease, and type of data for the model development (Fig. 2). The eligibility criteria were detailed, clear, and matched the review question in 13 reviews. Six studies showed a clear and adequate search strategy that contained all search terms related to the topic, Subject Headings, and limits. Less than half (*n* = 7) of the included reviews used adequate search sources such as searching multiple major databases and backward and forward reference list checking. Only five reviews assessed the quality of the included studies using a tool suitable for the review question. The quality assessment was carried out by two or more reviewers independently in only a single review. In three reviews, bias and errors in data extraction were minimal, given that at least two reviewers independently extracted the data using a piloted tool. Publication bias and its potential impact on the findings were assessed in only one review. All included reviews used an adequate approach for data synthesis and provided relevant research and practical implications based on the findings. Supplementary Table 1 shows reviewers’ judgments about each appraisal item for each included review.

### Results of studies

The included reviews assessed the performance of AI models in diagnosing 8 mental disorders: Alzheimer’s disease, mild cognitive impairment, schizophrenia, autism spectrum disorder, bipolar disease, obsessive-compulsive disorder, post-traumatic stress disorder, and psychotic disorders. The performance of the AI models in diagnosing these mental disorders is presented in the next subsections.

Alzheimer’s disease (AD) is a neurodegenerative disorder characterized by an ongoing decline in brain functions such as memory, executive functions, and language processing^32^. Four reviews assessed the performance of AI classifiers in differentiating AD from healthy control (HC) using neuroimaging data^17–20^ (Table 6). The number of mutual studies was five between Pellegrini et al.^17^ and Ebrahimighahnavieh et al.^20^ and four between Pellegrini et al.^17^ and Sarica et al.^19^. Accuracy, sensitivity, and specificity of the classifiers in these four reviews ranged from 56% to 100%, 37.3% to 100%, and 55% to 100%, respectively (Table 6). None of these reviews pooled the results using meta-analysis due to the high heterogeneity in the used classifiers, data types, data features, and types of validation.

**Table 6:** Classifier performance in differentiating AD from HC.

Study	AI approach	Accuracy (*n*)	Sensitivity (*n*)	Specificity (*n*)	AUC (*n*)
Neuroimaging data					
Pellegrini^17^	UML, SML, DL	71–98.1 (68)	60–99.2 (68)	75.9–98.3 (68)	NR
Billeci^18^	SML	56–100 (21)	37.3–100 (14)	55–100 (14)	NR
Sarica^19^	SML	87–98 (4)	NR	NR	NR
Ebrahimighahnavieh^20^	DL	75–100 (83)	73–100 (52)	80–100 (52)	NR
Neuropsychological data					
Petti^21^	UML, SML, DL	68–95 (17)	NR	NR	NR
Battista^22^	DL	72–100 (18)	73–100 (13)	77–100 (13)	79–98 (5)

*AD* Alzheimer’s disease, *AI* Artificial intelligence, *AUC* Area under the Curve, *DL* Deep learning, *HC* Healthy controls, *n* number of studies reported the corresponding measure, *SML* Supervised machine learning, *NR* not reported, *UML* Unsupervised machine learning.

Two other reviews examined the performance of AI classifiers in differentiating AD from HC using neuropsychological data^21,22^. There are four mutual studies between the two reviews. Accuracy of the classifiers in these reviews ranged from 68% to 100% (Table 6). One of these reviews meta-analyzed sensitivities and specificities reported in eleven studies and showed a pooled sensitivity of 92% and a pooled specificity of 86%^22^.

Three reviews examined the performance of AI classifiers in differentiating AD from mild cognitive impairment (MCI) using neuroimaging data^17,18,20^ (Table 7). There are five mutual studies between Pellegrini et al.^17^ and Ebrahimighahnavieh et al.^20^. Accuracy, sensitivity, and specificity of the classifiers in these three reviews ranged from 56% to 100%, 40.3% to 100%, and 67% to 100%, respectively (Table 7). None of these reviews pooled the results using meta-analysis due to the high heterogeneity. One other review examined the performance of AI classifiers in differentiating AD from MCI using neuropsychological data^21^. Accuracy of the classifiers in that review varied between 68% to 86% (Table 7).

**Table 7:** Classifier performance in differentiating AD from MCI.

Study	AI approach	Accuracy (*n*)	Sensitivity (*n*)	Specificity (*n*)	AUC (*n*)
Neuroimaging data					
Pellegrini^17^	UML, SML, DL	64.8–85.6 (8)	40.3–87 (8)	67–94.1 (8)	NR
Billeci^18^	SML	56–92 (6)	NR	NR	NR
Ebrahimighahnavieh^20^	DL	62.5–100 (27)	62.3–100 (15)	67.2–100 (15)	NR
Neuropsychological data					
Petti^21^	UML, SML, DL	68–86 (3)	NR	NR	NR

*AD* Alzheimer’s disease, *AI* Artificial intelligence, *AUC* Area under the Curve, *DL* Deep learning, *MCI* mild cognitive impairment, *n* number of studies reported the corresponding measure, *SML* Supervised machine learning, *NR* not reported, *UML* Unsupervised machine learning.

One review assessed the performance of AI classifiers in differentiating AD from Lewy body dementia (LBD) using EEG measures^23^. Accuracy, sensitivity, specificity, and AUC of the classifiers in this review ranged from 66% to 100%, 76% to 100%, 77% to 100%, and 78% to 93%, respectively.

Mild cognitive impairment (MCI) refers to deterioration in cognitive functions (e.g., memory, thinking, and language) that is detectable but it is less severe than the deterioration in patients with AD^33^. MCI represents a transitional stage between the expected cognitive decline associated with normal aging and the more severe decline of dementia^33^. Four reviews assessed the performance of AI classifiers in differentiating MCI from HC using neuroimaging data^17–20^ (Table 8). The number of mutual studies was five between Pellegrini et al.^17^ and Ebrahimighahnavieh et al.^20^ and four between Pellegrini et al.^17^ and Sarica et al.^19^. Accuracy, sensitivity, and specificity of the classifiers in these four reviews ranged from 47% to 99.2%, 24.3% to 98.3%, and 47.1% to 97%, respectively (Table 8). None of these reviews pooled the results using meta-analysis due to the high heterogeneity.

**Table 8:** Classifier performance in differentiating MCI from HC.

Study	AI approach	Accuracy (*n*)	Sensitivity (*n*)	Specificity (*n*)	AUC (*n*)
Neuroimaging data					
Pellegrini^17^	UML, SML, DL	61.8–92.7 (30)	49.5–94.8 (30)	47.3–90.8 (30)	NR
Billeci^18^	SML	47–97.7 (10)	24.3–95 (4)	66.4–97 (4)	NR
Sarica^19^	SML	58.4–82.3 (3)	NR	NR	NR
Ebrahimighahnavieh^20^	DL	55.2–99.2 (53)	52–98.3 (34)	47.1–95 (32)	NR
Neuropsychological data					
Petti^21^	UML, SML, DL	73–88.1 (4)	NR	NR	NR
Battista^22^	DL	60–98 (16)	45–97 (13)	67–100 (14)	63–99 (7)

*AI* Artificial intelligence, *AUC* Area under the Curve, *DL* Deep learning, *HC* Healthy controls, *MCI* mild cognitive impairment, *n* number of studies reported the corresponding measure, *SML* Supervised machine learning, *NR* not reported, *UML* Unsupervised machine learning.

Two other reviews examined the performance of AI classifiers in differentiating MCI from HC using neuropsychological data^21,22^. Four studies were mutual studies between the two reviews. Accuracy of the classifiers in these reviews ranged from 60% to 98% (Table 8). Only one of these reviews meta-analyzed sensitivities and specificities reported in nine studies and showed pooled sensitivity and specificity of 83% each^22^.

Three reviews examined the performance of AI classifiers in differentiating MCI converting to AD (MCIc) from MCI non-converting to AD (MCInc) using neuroimaging data^17,19,20^ (Table 9). The number of mutual studies was five between Pellegrini et al.^17^ and Ebrahimighahnavieh et al^20^ and four between Pellegrini et al.^17^ and Sarica et al.^19^. Accuracy, sensitivity, and specificity of the classifiers in these three reviews ranged from 47% to 96.2%, 42.1% to 99%, and 51.2% to 95.2%, respectively (Table 10). None of these reviews pooled the results using meta-analysis due to the high heterogeneity.

**Table 9:** Classifier performance in differentiating MCIc from MCInc.

Study	AI approach	Accuracy (*n*)	Sensitivity (*n*)	Specificity (*n*)	AUC (*n*)
Neuroimaging data					
Pellegrini^17^	UML, SML, DL	56.1–82.5 (38)	56.2–94.2 (38)	51.2–89 (38)	NR
Sarica^19^	SML	58.4–82.3 (4)	NR	NR	NR
Ebrahimighahnavieh^20^	DL	47–96.2 (27)	42.1–99 (19)	53–95.2 (19)	NR
Neuropsychological data					
Battista^22^	DL	61–85 (19)	50–91 (16)	48–91 (16)	67–93 (14)

*AI* Artificial intelligence, *AUC* Area under the Curve, *DL* Deep learning, *MCIc* MCI converting, *MCInc* MCI non-converting, *n* number of studies reported the corresponding measure, *SML* Supervised machine learning, *NR* not reported, *UML* Unsupervised machine learning.

**Table 10:** Classifier performance in differentiating SCZ from HC.

Study	AI approach	Accuracy (*n*)	Sensitivity (*n*)	Specificity (*n*)	AUC (*n*)
Neuroimaging data					
de Filippis^24^	UML, SML, DL	61–99.3 (28)	57.9–100 (20)	40.9–98.6 (20)	NR
Steardo^25^	SML	61–99.3 (22)	65–100 (17)	40.9–98.6 (17)	61–91.4 (3)
Genetic data					
Bracher-Smith^26^	DL	40–86 (5)	NR	NR	54–95 (5)

*AI* Artificial intelligence, *AUC* Area under the Curve, *DL* Deep learning, *HC* Healthy controls, *n* number of studies reported the corresponding measure, *SCZ* Schizophrenia, *SML* Supervised machine learning, *NR* not reported, *UML* Unsupervised machine learning.

Another review examined the performance of AI classifiers in differentiating MCIc from MCInc using neuropsychological data^22^. Accuracy, sensitivity, specificity, and AUC of the classifiers in this review ranged from 61% to 85%, 50% to 91%, 48% to 91%, and 67% to 93%, respectively. This review meta-analyzed sensitivities and specificities reported in ten studies and showed a pooled sensitivity of 73% and a pooled specificity of 69%.

Schizophrenia (SCZ) is a long-term serious mental disorder, in which patients are not able to differentiate between their thoughts from reality due to disturbances in cognition, emotional responsiveness, and behavior^34^. Two reviews investigated the performance of AI classifiers in differentiating SCZ from HC using neuroimaging data^24,25^. There are 15 mutual studies between the two reviews. Accuracy, sensitivity, and specificity of the classifiers in the two reviews ranged from 61% to 99.3%, 57.9% to 100%, and 40.9% to 98.6%, respectively (Table 10). None of these reviews pooled the results using meta-analysis. One review examined the performance of AI classifiers in differentiating SCZ from HC using genetic data^26^. Accuracy and AUC of the classifiers in this review ranged from 40% to 86% and 54% to 95%, respectively.

Bipolar disorder is a mood disorder that is characterized by mood fluctuations between symptoms of mania or hypomania and depression^35^. One review assessed the performance of AI classifiers in differentiating bipolar BD from HC using neuroimaging data^27^. Accuracy, sensitivity, and specificity of the classifiers ranged from 55% to 100%, 40% to 100%, and 49% to 100%, respectively (Table 11). This review examined the performance of AI classifiers in differentiating BD from HC using neuropsychological data^27^. Accuracy of classifiers varied between 71% and 96.4% (Table 11). This review also investigated the performance of AI classifiers in differentiating BD from major depressive disorder using neuroimaging data. Accuracy, sensitivity, and specificity of the classifiers ranged from 54.76% to 92.1% (*n* = 7), 57.9 to 83% (*n* = 3), and 52.1 to 90.9% (*n* = 3), respectively. Another review used genetic data and AI classifiers to differentiate BD from HC^26^. Accuracy and AUC of the classifiers ranged from 54% to 77% and 48% to 65%, respectively (Table 11).

**Table 11:** Classifier performance in differentiating BD from HC.

Study	AI approach	Accuracy (*n*)	Sensitivity (*n*)	Specificity (*n*)	AUC (*n*)
Neuroimaging data					
Librenza-Garcia^27^	UML, SML, DL	55–100 (8)	40–100 (12)	49–100 (12)	NR
Neuropsychological data					
Librenza-Garcia^27^	UML, SML, DL	71–96.4 (3)	NR	NR	NR
Genetic data					
Bracher-Smith^26^	UML, SML, DL	54–77 (4)	NR	NR	48–65 (3)

*AI* Artificial intelligence, *AUC* Area under the Curve, *BD* bipolar disorders, *DL* Deep learning; *HC* Health control, *n* number of studies reported the corresponding measure, *SML* Supervised machine learning, *NR* not reported, *UML* Unsupervised machine learning.

Autism spectrum disorder (ASD) is a group of disorders (e.g., autism, childhood disintegrative disorder, and Asperger’s disorder) that starts usually in the preschool period and is characterized by difficulties or impairment in communication and social interaction^36^. One review investigated the performance of AI classifiers in differentiating ASD from HC using neuroimaging data^28^. Accuracy, sensitivity, and specificity of the classifiers in the review ranged from 45% to 97%, 24% to 100%, and 21% to 100%, respectively (Table 12). The review meta-analyzed sensitivities and specificities of AI classifiers based on structured MRI (sMRI) in 11 studies. The review found a pooled sensitivity of 83%, a pooled specificity of 84%, a pooled AUC of 90%^28^. The review also meta-analyzed sensitivities and specificities of deep neural network-based classifiers in one study (five samples) that used functional MRI (fMRI) as a predictor. The review found a pooled sensitivity of 69%, a pooled specificity of 66%, and a pooled AUC of 71%^28^.

**Table 12:** Classifier performance in differentiating ASD from HC.

Study	AI approach	Accuracy (*n*)	Sensitivity (*n*)	Specificity (*n*)	AUC (*n*)
Neuroimaging data					
Moon^28^	UML, SML, DL	45–97 (20)	24–100 (20)	21–99 (20)	NR
Neuropsychological tests					
Moon^28^	UML, SML, DL	78.1–100 (9)	64–100 (9)	48–97 (9)	NR
Biochemical features					
Moon^28^	UML, SML, DL	75–94 (5)	77–94 (5)	67–93 (5)	NR
EEG measures					
Moon^28^	UML, SML, DL	85–100 (4)	94–97 (4)	81–94 (4)	NR

*AI* Artificial intelligence, *ASD* autism spectrum disorder, *AUC* Area under the Curve, *DL* Deep learning, *HC* Healthy control, *SML* Supervised machine learning, *n* number of studies reported the corresponding measure, *NR* not reported, *UML* Unsupervised machine learning.

The review assessed the performance of AI classifiers in differentiating ASD from HC using a neuropsychological test (behavior traits)^28^. Accuracy, sensitivity, and specificity of the classifiers in the review ranged from 78.1% to 100%, 64% to 100%, and 48% to 97%, respectively (Table 12). Further, the review tested the performance of AI classifiers in differentiating ASD from HC using biochemical features^28^. Accuracy, sensitivity, and specificity of the classifiers in the review ranged from 75% to 94%, 77% to 94%, and 67% to 93%, respectively (Table 12). The review also examined the performance of AI classifiers in differentiating ASD from HC using EEG measures^28^. Accuracy, sensitivity, and specificity of the classifiers in the review ranged from 85% to 100%, 94% to 97%, and 81% to 94%, respectively (Table 12). The review did not conduct a meta-analysis for the above-mentioned results due to heterogeneity between samples^28^.

Posttraumatic stress disorder (PTSD) refers to feelings of fear, anxiety, irritability, terror, or guilty that result from remembering very stressful, life-threatening, frightening, distressing events that a patient lived through or witnessed in the past^37^. One review examined the performance of AI classifiers in differentiating PTSD from HC^29^. Accuracy of the classifiers using neuroimaging data varied between 89.2% and 92.3% (*n* = 3). The review also assessed the performance of AI classifiers in differentiating PTSD from trauma-exposed controls^29^. Accuracy of the classifiers using neuroimaging data varied between 67% and 83.6% (*n* = 4). Meta-analysis was not carried out in the review.

Obsessive-compulsive disorder (OCD) is a mental health condition in which an individual has frequent intrusive thoughts that lead him or her to perform repetitive behaviors, which may affect daily activities and cause severe distress^38^. One review assessed the performance of supervised machine learning classifiers in distinguishing OCD from HC using neuroimaging data^30^. Accuracy, sensitivity, and specificity of the classifiers in the review ranged from 66% to 100% (*n* = 11), 74.1% to 96.2% (*n* = 6), and 72.7% to 95% (*n* = 6), respectively. The review did not pool the results using meta-analysis.

Psychotic disorders are a group of mental disorders in which a patient has incorrect perceptions, thoughts, and inferences about external reality although there is contrary evidence^39^. One review examined the performance of AI classifiers in differentiating patients with a high risk of developing psychotic disorders from HC using neuroimaging data or neuropsychological tests^31^. Sensitivity and specificity of the classifiers in the review ranged from 60% to 96% (*n* = 12) and 47% to 94 (*n* = 12), respectively. The review meta-analyzed sensitivities and specificities of AI classifiers in 12 studies and found a pooled sensitivity of 78% and a pooled specificity of 77%^31^.

## Discussion

This umbrella review provides an evidence map of the state of the art of AI technologies in diagnosing mental health disorders. The 15 included systematic reviews focused on diagnosing 8 mental disorders. Considering the probability for MCI to progress into clinically diagnosed AD paired with our still limited understanding of contributing factors, it is hardly surprising that more than 200 original studies and 40% of the included reviews focused on AD and MCI.

We also observe that the reported pooled sensitivity of 92% and specificity of 86% for classifying AD vs. HC is higher than for classifying MCI vs. HC (83% pooled sensitivity and specificity), and both are higher than for classifying MCIc vs. MCInc (73% pooled sensitivity and 69% specificity)^22^. This may be attributed to the fact that AD is a neurodegenerative disease, thereby, there is a continuum ranging from AD on one extreme to HC on the other. Accordingly, discerning extremal cases seems intuitively easier than between more similar stages. This is in line with the reported performances for differentiating PTSD from HC being higher than from trauma-exposed controls^29^. However, we would also like to point out that the same review reports methods with better performance than the pooled sensitivities and specificities quoted above. This raises the question if such pooling is meaningful from the point of a user, since it obfuscates the existence of better diagnostic tools in the same review.

For classifying SCZ vs. HC, we observe that neuroimaging data tends to lead to better-performing classifiers than genetic data. Unsurprisingly, using genetic data alone leads to significantly lower performance, reflecting that both genetic and environmental factors causing SCZ are described in the literature^40^. Likewise, classifying BD from HC using genetic data alone shows lower performance. It is interesting to note that for BD vs. HC, neuropsychological data seems to achieve decent accuracy (71%-96.4%) more reliably than neuroimaging data (55%-100%). However, this may also be a result of low sample count (*n* = 3 using neuropsychological data, *n* = 8 using neuroimaging data).

For discriminating ASD from HC, most data types can support methods with good accuracy but using biochemical features or EEG measures lead to a significantly increased sensitivity and specificity. Structured MRI leads to better-pooled specificities and sensitivities when compared to functional MRI. This can be attributed to two reasons: (1) sMRI findings resulted from pooling 12 samples from 10 different studies while fMRI resulted from five samples from only two studies, and (2) the deep neural network (DNN) was used as a classifier in the fMRI studies whereas it was used as a classifier in only one sMRI study^28^.

One review showed promising results regarding the performance of AI models in distinguishing OCD from HC using neuroimaging data. These results should be interpreted carefully for three reasons. First, these results are based on studies with small samples (i.e., 20-172). Second, most included studies used cross validation methods to assess the performance of their models, which is not the most suitable method when the sample size is small. Third, large heterogeneity in OCD patients and the classification features in the included studies.

We found acceptable pooled sensitivity (78%) and pooled specificity (77%) for differentiating patients with a high risk of developing psychotic disorders from HC. However, the authors of that review could not draw a definitive conclusion about applicability of AI models due to high clinical and methodological heterogeneity in meta-analyzed studies.

Reporting practices in the original literature continue to severely hinder statistical meta-analysis of results. On the one hand, the reported up-to-perfect performance for many tasks by the included studies signals a new age of AI, where, given the right modality and amount of data impressive results are reported tasks with real-world significance. However, considering that many original studies seemingly choose performance metrics at random could suggest a definition of success by choice of metric rather than by the task at hand. This, in turn, leaves us with an ambivalent feeling regarding the usefulness of attempts of such analyses (as, e.g., performed by Battista et al.^22^). Between two competing methods that (a) are properly validated with a large enough cohort, (b) have shown sufficient generalization (e.g., in the form of an external validation) and that (c) use the same data modality, the one with the better performance should be chosen. This underscores the importance of following proper reporting practices, since statistical evaluation (from a clinical, not technological point of view) otherwise seems moot.

The included reviews focused on the performance of AI models in diagnosing 8 mental disorders. However, our search process did not pick up on systematic reviews for several other mental disorders, such as major depressive disorder (MDD), anxiety, eating disorders, and personality disorders. Thus, there is a need to conduct systematic reviews to synthesize the evidence on performance of AI models in diagnosing such mental disorders.

The systematic review of AI studies differentiating high-risk psychosis cases with healthy controls^31^ is a case example of where the field could benefit from more research. The benefits of early diagnosis could offer the opportunity for intervention prior to full development of a psychotic disorder. Further studies could focus on at-risk groups or identifying ‘at-risk’ for other disorders such as anxiety and MDD and possibly broaden data source types to those that are more accessible and practical than neuroimaging data.

Neuroimaging data for AI models seemed to dominate in the systematic reviews included in this review. In spite of the promising performance of these AI models, we question the practicality of incorporating neuroimaging data into routine diagnostic practice due to it being a resource-intensive procedure. By contrast, AI models of neuropsychological, genetic, and EEG tests could offer exciting opportunities to complement and improve existing diagnostic processes in mental healthcare.

According to the performance reported in the included studies, AI shows a great potential to lead to accelerated, accurate, and more objective diagnoses. The findings in this review strongly suggest that AI is on the jump into clinical use. We believe it is therefore important to educate practitioners exploring the potential for new diagnostic and therapeutic methods as they shift their focus as in so many other jobs that now begin utilizing AI^8^; this exploratory use should be ethical and cautious. The availability of high-quality AI solutions may even pave the way for an entirely new medical specialization. More important for reliable AI-based classifiers than sample sizes, however, are reproducibility and generality. For a method to be reproducible, data and code must be made available, such that other research teams can verify the code and ensure that the method is free from oversights. For a method to be general, it must deliver results similar to the reported ones on new, previously unseen data. Currently, single site cross-validation is the most common approach; however, validation of new models would benefit greatly from replication using data from external samples.

Many original studies focus on the technical/algorithmic aspects rather than the choice of data modality. This is a consequence of the fact that (supervised) AI is extremely data-hungry, yet high-quality, labeled data is a scarce and expensive resource. It represents a significant amount of effort and manpower. This dependence of contemporary AI on humans dedicating time to first gather and clean, then feed it with data has been likened to a parasitic relationship^41,42^. As the AI grows, it promises higher utility to humans, which are thus motivated to sift through more data. The temptation to achieve results with the data at hand instead of a thorough investigation into which modality offers the best results is understandably high.

The main limitation of this review is that the data was not synthesized statistically. We could not synthesize the data statistically for three reasons. Firstly, the included reviews were inconsistent in reporting the results of classifier performance. Secondly, most reviews did not extract or present data that is necessary for assessing classifier performance and aggregating the data statistically (i.e., true positive, false positive, true negative, and false negative). Lastly and most importantly, there was high heterogeneity in the AI classifiers (e.g., SVM, DT, RF, CNN, K-NN), data types (e.g., neuroimaging data, genetic data, demographic data), data features (e.g., axial diffusivity, radial diffusivity, mean diffusivity, fractional anisotropy), target mental disorder, model validation approach, and measures of classifier performance reported in the included reviews.

We also do not present the range of performance metrics for classification tasks that were reported by less than three studies. For example, we do not report the classifier performance of AI approaches in distinguishing anorexia nervosa from healthy controls as it was assessed by only one study in one of the included reviews^26^. Another limitation of this review is that we did not exclude the mutual primary studies between reviews. Therefore, there may be some duplicates in the ranges of classifier performance reported in our review. However, we declared the number of mutual studies between reviews when we aggregated ranges from more than two reviews. We did not exclude reviews based on their quality because most included reviews were judged as low quality in at least four appraisal items. Quality-based exclusion would therefore have resulted in including too few reviews in this work.

To conclude, AI shows a great potential to lead to accelerated, accurate, and more objective diagnoses of mental health disorders. The findings in this review strongly suggest that AI is on the jump into clinical use. Up-to-perfect performance is reported in many of the included studies, but much of that performance depends on the correct choice of data modality paired with correct technical choices (e.g., AI algorithms and methods). While AI promises a valid path for impartial and objective classification of mental disorders, practitioners in any field need to understand the basic aspect and behavior of their tools. We therefore believe that ethical considerations will gain importance in the future as well. With these considerations in mind, we recommend that healthcare professionals in the field (e.g., psychiatrists, psychologists) cautiously and consciously begin to explore the opportunities of AI-based tools for their daily routine. This recommendation is based on the potential we see in the technology reviewed in this study and the hope for rigorous evaluation in a clinical environment.

## Methods

An umbrella review was conducted and reported in keeping with the Joanna Briggs Institute’s (JBI) guidelines for umbrella reviews^43^. The protocol for this review is registered at PROSPERO (ID: CRD42021231558).

### Search strategy

We utilized the following bibliographic databases in our search: MEDLINE (via Ovid), PsycInfo (via EBSCO), CINAHL (EBSCO), IEEE Xplore, ACM Digital Library, Scopus, Cochrane Database of Systematic Reviews, DARE, and the PROSPERO register, JBI Evidence Synthesis, and Epistemonikos. These databases were searched on August 12, 2021 by the lead author. When applicable, we set auto alerts to conduct an automatic search weekly for 12 weeks (ending on December 12, 2021). We also searched the search engine “Google Scholar” to identify gray literature. We checked only the first 50 hits given that Google Scholar retrieved a massive number of hits and order them based on their relevancy. To identify further studies of relevance to the review, we screened the reference lists of included reviews (i.e., backward reference list checking) and identified and screened systematic reviews that cited the included reviews (i.e., forward reference list checking).

We developed the search query by consulting two experts in digital mental health and by checking systematic reviews of relevance to the review. These terms were chosen based on the target population (i.e., mental disorders), target intervention (i.e., AI-based approaches), and target study design (i.e., systematic review). Supplementary Table 2 presents the detailed search query used for searching each database.

### Study eligibility criteria

This review included systematic reviews that focused on the performance of AI-based approaches in diagnosing mental disorders regardless of data type (e.g., neuroimaging data, neuropsychological data, demographical data, and clinical data), year of publication, and country of publication. We excluded systematic reviews that focused on AI-based approaches for predicting outcomes of intervention or prognosis of mental disorders. We also excluded reviews that did not show at least one of the following measures of classifier performance: accuracy, sensitivity, specificity, or area under the curve (AUC). Further, we excluded primary studies, scoping reviews, literature reviews, rapid reviews, criterial reviews, and other types of reviews. While systematic reviews published as journal articles, conference proceedings, and dissertations were included, we excluded conference abstracts and posters, commentaries, preprints, proposals, and editorials. We considered systematic reviews published only in the English language.

### Study selection

We followed two steps to identify the relevant reviews. In the first step, two reviewers (AA and MH) independently checked the titles and abstracts of all identified studies. In the second step, the full texts of studies included from the first step were read by the two reviewers independently. In both steps, the two reviewers resolved any disagreements through discussion and consensus.

### Data extraction

We developed a form to precisely and systematically extract the data from the included reviews (Supplementary Table 3). The form was pilot-tested using two included reviews. Two reviewers (AA & MH) independently extracted data from the included reviews using Microsoft Excel. Any disagreements between the reviewers were resolved through discussion and consensus.

### Study quality appraisal

Two reviewers (AA and MH) independently assessed the quality of the included reviews using Joanna Briggs Institute Critical Appraisal Checklist for Systematic Reviews and Research Syntheses^43^. Any disagreements between the reviewers were resolved through discussion and consensus. Inter-rater agreement between the reviewers was very good (0.85)^44^.

### Data synthesis

We synthesized the extracted data using the narrative approach. Specifically, results of the included reviews were grouped based on the target mental disorders that the AI classifiers distinguish. The results in each group were further aggregated based on the data types used to diagnose the target mental disorder. Given the high heterogeneity in the AI classifiers, data types, target mental disorder, and measures of classifier performance reported in the included reviews, we could not synthesize the results statistically. Therefore, we reported the range of results of measures of classifier performance. In addition, results that were reported by fewer than three primary studies in the included reviews are not reported in our review.

### Reporting summary

Further information on research design is available in the Nature Research Reporting Summary linked to this article.

## Supplementary information

Reporting Summary

Supplementary information

## Data Availability

The data that support the findings of this study are available from the corresponding author upon reasonable request.
