# Supplementary material for: The performance of artificial intelligence-driven technologies in diagnosing mental disorders: an umbrella review
Source: NPJ Digit Med. 2022 Jul 7;5:87. doi: 10.1038/s41746-022-00631-8 (PMC9262920; doi:10.1038/s41746-022-00631-8)
Supplement: Supplementary file 2 — Supplementary information [file 41746_2022_631_MOESM2_ESM.pdf]

Supplementary Table 1: Reviewers' judgements about each quality criterion for each included review

	Clear question	Appropriate criteria	Appropriate search strategy	Adequate search sources	Appropriate criteria for quality appraisal	≥2 reviewers for quality appraisal	Appropriate data extraction	Appropriate data synthesis	Assessment of publication bias	Appropriate practical implications	Appropriate research implications
Pellegrini [1]	No	Yes	Unclear	No	Yes	Unclear	Unclear	Yes	NA	Yes	Yes
Billeci [2]	Yes	Yes	Unclear	No	No	NA	Unclear	Yes	NA	Yes	Yes
Sarica [3]	Yes	Yes	Unclear	No	No	NA	Unclear	Yes	NA	Yes	Yes
Ebrahimighahnavieh [4]	No	Yes	Unclear	Yes	No	Unclear	Unclear	Yes	NA	Yes	Yes
Petti [5]	Yes	Yes	No	No	No	NA	Unclear	Yes	NA	Yes	Yes
Battista [6]	Yes	Yes	Unclear	Unclear	Yes	Unclear	Unclear	Yes	No	Yes	Yes
Law [7]	Yes	Yes	Yes	Yes	Yes	Unclear	Yes	Yes	NA	Yes	Yes
de Filippis [8]	No	Yes	Unclear	Yes	No	Unclear	Unclear	Yes	NA	Yes	Yes
Steardo [9]	Yes	No	Unclear	Yes	No	Unclear	Unclear	Yes	NA	Yes	Yes
Bracher-Smith [10]	Yes	Yes	Yes	No	Yes	No	Yes	Yes	NA	Yes	Yes
Librenza-Garcia [11]	No	Yes	Yes	Yes	No	NA	Unclear	Yes	No	Yes	Yes
Moon [12]	No	Yes	Yes	No	Yes	Yes	Yes	Yes	No	Yes	Yes
Ramos-Lima [13]	Yes	Yes	Yes	Yes	No	Unclear	Unclear	Yes	NA	Yes	Yes
Bruin [14]	Yes	Unclear	Unclear	No	No	NA	Unclear	Yes	NA	Yes	Yes
Sanfelici [15]	Yes	Yes	Yes	Yes	No	NA	Unclear	Yes	Yes	Yes	Yes

1. Pellegrini E, Ballerini L, Hernandez M, Chappell FM, Gonzalez-Castro V, Anblagan D, et al. Machine learning of neuroimaging for assisted diagnosis of cognitive impairment and dementia: A systematic review. Alzheimer's & Dementia : Diagnosis, Assessment & Disease Monitoring. 2018;10:519-35. PMID: 30364671.
2. Billeci L, Badolato A, Bachi L, Tonacci A. Machine learning for the classification of alzheimer's disease and its prodromal stage using brain diffusion tensor imaging data: A systematic review. 2020;8(9). doi: 10.3390/pr8091071.

3. Sarica A, Cerasa A, Quattrone A. Random Forest Algorithm for the Classification of Neuroimaging Data in Alzheimer's Disease: A Systematic Review. Frontiers in aging neuroscience. 2017;9:329. PMID: 29056906.
4. Ebrahimighahnavieh MA, Luo S, Chiong R. Deep learning to detect Alzheimer's disease from neuroimaging: A systematic literature review. Computer Methods & Programs in Biomedicine. 2020;187:105242. PMID: 31837630.
5. Petti U, Baker S, Korhonen A. A systematic literature review of automatic Alzheimer's disease detection from speech and language. Journal of the American Medical Informatics Association. 2020;27(11):1784-97. doi: 10.1093/jamia/ocaa174.
6. Battista P, Salvatore C, Berlingeri M, Cerasa A, Castiglioni I. Artificial intelligence and neuropsychological measures: The case of Alzheimer's disease. Neuroscience & Biobehavioral Reviews. 2020;114:211-28. PMID: 32437744.
7. Law ZK, Todd C, Mehraram R, Schumacher J, Baker MR, LeBeau FEN, et al. The Role of EEG in the Diagnosis, Prognosis and Clinical Correlations of Dementia with Lewy Bodies-A Systematic Review. Diagnostics. 2020;10(9):20. PMID: 32825520. doi: 10.3390/diagnostics10090616.
8. de Filippis R, Carbone EA, Gaetano R, Bruni A, Pugliese V, Segura-Garcia C, et al. Machine learning techniques in a structural and functional MRI diagnostic approach in schizophrenia: a systematic review. Neuropsychiatric Disease & Treatment. 2019;15:1605-27. PMID: 31354276.
9. Steardo L, Jr., Carbone EA, de Filippis R, Pisanu C, Segura-Garcia C, Squassina A, et al. Application of Support Vector Machine on fMRI Data as Biomarkers in Schizophrenia Diagnosis: A Systematic Review. Frontiers in psychiatry Frontiers Research Foundation. 2020;11:588. PMID: 32670113.
10. Bracher-Smith M, Crawford K, Escott-Price V. Machine learning for genetic prediction of psychiatric disorders: a systematic review. Molecular Psychiatry. 2020;26:26. PMID: 32591634.
11. Librenza-Garcia D, Kotzian BJ, Yang J, Mwangi B, Cao B, Pereira Lima LN, et al. The impact of machine learning techniques in the study of bipolar disorder: A systematic review. Neuroscience & Biobehavioral Reviews. 2017;80:538-54. PMID: 28728937.
12. Moon SJ, Hwang J, Kana R, Torous J, Kim JW. Accuracy of Machine Learning Algorithms for the Diagnosis of Autism Spectrum Disorder: Systematic Review and Meta-Analysis of Brain Magnetic Resonance Imaging Studies. JMIR Mental Health. 2019;6(12):e14108. PMID: 31562756. doi: doi: 10.2196/14108.
13. Ramos-Lima LF, Waikamp V, Antonelli-Salgado T, Passos IC, Freitas LHM. The use of machine learning techniques in trauma-related disorders: a systematic review. Journal of Psychiatric Research. 2020;121:159-72. PMID: 31830722.
14. Bruin W, Denys D, van Wingen G. Diagnostic neuroimaging markers of obsessive-compulsive disorder: Initial evidence from structural and functional MRI studies. Progress in Neuropsychopharmacology & Biological Psychiatry. 2019;91:49-59. doi: 10.1016/j.pnpbp.2018.08.005.
15. Sanfelici R, Dwyer DB, Antonucci LA, Koutsouleris N. Individualized Diagnostic and Prognostic Models for Patients With Psychosis Risk Syndromes: A Meta-analytic View on the State of the Art. Biological Psychiatry. 2020;88(4):349-60. PMID: 32305218.

Supplementary Table 2: Search query

Database(s): **Ovid MEDLINE(R) ALL** 1946 to August 12, 2021
Search Strategy:

#	Searches	Results
1	exp Mental Health/	40895
2	mental health.tw.	151759
3	exp Mental Disorders/	1262958
4	"mental disorder*".tw.	40304
5	exp Mood Disorders/	123750
6	"mood disorder*".tw.	17560
7	exp Anxiety Disorders/	80440
8	"anxiety disorder*".tw.	31482
9	exp Depressive Disorder/	110670
10	"depressive disorder".tw.	29221
11	exp Bipolar Disorder/	40830
12	"Bipolar disorder*".tw.	28923
13	exp Psychotic Disorders/	52805
14	"psychotic disorder*".tw.	9048
15	exp Obsessive-Compulsive Disorder/	14824
16	"obsessive-compulsive disorder*".tw.	13592
17	exp Panic Disorder/	6949
18	"panic disorder*".tw.	9591
19	exp Phobic Disorders/	11427
20	"phobic disorder*".tw.	411
21	exp Substance-Related Disorders/	282449
22	"substance-related disorder*".tw.	555
23	exp Personality Disorders/	41690
24	"Personality disorder*".tw.	20353
25	exp Stress Disorders, Post-Traumatic/	33628
26	"post-traumatic stress disorder*".tw.	11947
27	exp Reactive Attachment Disorder/	572
28	"Attachment disorder*".tw.	298
29	exp "Feeding and Eating Disorders"/	31217
30	"eating disorder*".tw.	20148
31	exp Sleep Wake Disorders/	91942
32	"Sleep disorder*".tw.	19957

33	exp "Attention Deficit and Disruptive Behavior Disorders"/	33517
34	"Disruptive Disorder*".tw.	217
35	exp Adjustment Disorders/	4233
36	"Adjustment Disorder*".tw.	1756
37	exp Dissociative Disorders/	4327
38	"Dissociative disorder*".tw.	863
39	exp Somatoform Disorders/	19175
40	"Somatic symptom disorder*".tw.	264
41	"somatoform disorder*".tw.	1948
42	exp Neurocognitive Disorders/	261034
43	"Neurocognitive Disorder*".tw.	2600
44	exp Delirium/	9860
45	Delirium.tw.	15830
46	anxious.tw.	17209
47	phobi*.tw.	11650
48	exp Depression/	123027
49	depression.tw.	343509
50	depressed.tw.	94969
51	melancholia.tw.	1411
52	Mania.tw.	10563
53	exp Autistic Disorder/	20730
54	Autistic Disorder.tw.	1295
55	autism.tw.	45967
56	exp Attention Deficit Disorder with Hyperactivity/	29425
57	"Attention-Deficit Hyperactivity Disorder".tw.	25482
58	bipolar.tw.	64271
59	schizophrenia.tw.	109544
60	Stress.tw.	768150
61	exp Factitious Disorders/	3068
62	"Factitious Disorder*".tw.	550
63	paranoia.tw.	2152
64	psychosis.tw.	37684
65	anorexia.tw.	30124
66	binge-eating.tw.	5871
67	bulimia.tw.	7532
68	"drug dependence".tw.	3861
69	"substance dependence".tw.	2253

70	addiction.tw.	44982
71	"drug abuse".tw.	17023
72	"substance abuse".tw.	24408
73	"Narcolepsy".tw.	4626
74	"Insomnia".tw.	21780
75	"Hypersomnolence".tw.	626
76	Parasomnias.tw.	818
77	Kleptomania.tw.	243
78	Pyromania.tw.	88
79	"Intermittent explosive disorder".tw.	292
80	exp "Disruptive, Impulse Control, and Conduct Disorders"/	9026
81	"Oppositional Defiant Disorder".tw.	2022
82	"Alzheimer's disease".tw.	121148
83	"Asperger syndrome".tw.	1012
84	Dementia.tw.	111249
85	"Parkinson's disease".tw.	85864
86	"Borderline personality disorder".tw.	6499
87	1 or 2 or 3 or 4 or 5 or 6 or 7 or 8 or 9 or 10 or 11 or 12 or 13 or 14 or 15 or 16 or 17 or 18 or 19 or 20 or 21 or 22 or 23 or 24 or 25 or 26 or 27 or 28 or 29 or 30 or 31 or 32 or 33 or 34 or 35 or 36 or 37 or 38 or 39 or 40 or 41 or 42 or 43 or 44 or 45 or 46 or 47 or 48 or 49 or 50 or 51 or 52 or 53 or 54 or 55 or 56 or 57 or 58 or 59 or 60 or 61 or 62 or 63 or 64 or 65 or 66 or 67 or 68 or 69 or 70 or 71 or 72 or 73 or 74 or 75 or 76 or 77 or 78 or 79 or 80 or 81 or 82 or 83 or 84 or 85 or 86	2607716
88	exp Artificial Intelligence/	105229
89	"Artificial intelligence".tw.	9571
90	exp Machine Learning/	23072
91	"Machine learning".tw.	33867
92	exp Deep Learning/	3832
93	"deep learning".tw.	12582
94	exp Neural Networks, Computer/	30455
95	"Neural network*".tw.	54107
96	exp Supervised Machine Learning/	7819
97	"supervised learning".tw.	2651
98	exp Unsupervised Machine Learning/	394
99	"unsupervised learning".tw.	1262
100	exp Natural Language Processing/	4442
101	88 or 89 or 90 or 91 or 92 or 93 or 94 or 95 or 96 or 97 or 98 or 99 or 100	163289

102	exp "Systematic Review"/	142470
103	"systematic review".tw.	174439
104	"systematic literature review".tw.	12029
105	exp Meta-Analysis/	124597
106	"meta-analysis".tw.	163391
107	102 or 103 or 104 or 105 or 106	301791
108	87 and 101 and 107	223
109	limit 108 to english language	220

Database(s): **CINAHL Plus with Full Text (EBSCO)**
Search Strategy:

#	Query	Results
S1	MH "mental health"	40,322
S2	AB "mental health"	88,616
S3	MH "mental disorder*"	64,450
S4	AB "mental disorder*"	12,999
S5	AB "mood disorder*"	4,479
S6	MH "anxiety disorder*"	10,781
S7	AB "anxiety disorder*"	9,488
S8	MH "depressive disorder*"	0
S9	AB "depressive disorder*"	9,744
S10	MH "Bipolar disorder*"	12,112
S11	AB "Bipolar disorder*"	7,292
S12	MH "psychotic disorder*"	12,824
S13	AB "psychotic disorder*"	2,960
S14	MH "obsessive-compulsive disorder*"	5,093
S15	AB "obsessive-compulsive disorder*"	2,976
S16	MH "panic disorder*"	2,307
S17	AB "panic disorder*"	1,695

S18	MH "phobic disorder*"	2,462
S19	MH "phobic disorder*"	2,462
S20	MH "substance-related disorder*"	0
S21	AB "substance-related disorder*"	210
S22	MH "Personality disorder*"	4,605
S23	AB "Personality disorder*"	6,198
S24	MH "post-traumatic stress disorder*"	0
S25	AB "post-traumatic stress disorder*"	597
S26	MH "Attachment disorder*"	0
S27	AB "Attachment disorder*"	145
S28	MH "eating disorder*"	9,922
S29	AB "eating disorder*"	9,278
S30	MH "Sleep disorder*"	12,233
S31	AB "Sleep disorder*"	5,504
S32	MH "Disruptive Disorder*"	0
S33	AB "Disruptive Disorder*"	79
S34	MH "Adjustment disorder*"	649
S35	AB "Adjustment disorder*"	477
S36	MH "Dissociative disorder*"	1,488
S37	AB "Dissociative disorder*"	213
S38	MH "Somatic symptom disorder*"	0
S39	AB "Somatic symptom disorder*"	100
S40	MH "somatoform disorder*"	3,720
S41	AB "somatoform disorder*"	443
S42	MH "Neurocognitive Disorder*"	0
S43	AB "Neurocognitive Disorder*"	575
S44	MH "Autistic Disorder*"	24,866
S45	AB "Autistic Disorder*"	467

S46	MH "Factitious Disorder*"	293
S47	AB "Factitious Disorder*"	113
S48	MH Delirium	6,825
S49	AB Delirium	6,092
S50	AB anxious	5,606
S51	AB phobi*	2,397
S52	MH depression	110,844
S53	AB depression	107,337
S54	AB melancholia	140
S55	AB Mania	2,054
S56	AB autism	17,994
S57	MH "Attention-Deficit Hyperactivity Disorder"	0
S58	AB "Attention-Deficit Hyperactivity Disorder"	5,526
S59	AB bipolar	12,048
S60	AB schizophrenia	21,249
S61	AB Stress	123,973
S62	AB paranoia	663
S63	AB psychosis	10,570
S64	AB anorexia	6,619
S65	AB binge-eating	2,644
S66	AB bulimia	2,618
S67	AB "drug dependence"	934
S68	AB "substance dependence"	821
S69	AB addiction	15,832
S70	AB "drug abuse"	4,257
S71	AB "substance abuse"	11,584
S72	AB "Narcolepsy"	831
S73	AB Insomnia	7,205

S74	AB Hypersomnolence	142
S75	AB Parasomnias	357
S76	AB Kleptomania	42
S77	AB Pyromania	12
S78	AB "Intermittent explosive disorder"	64
S79	AB "Oppositional Defiant Disorder"	840
S80	AB "Alzheimer's disease"	19,379
S81	AB "Asperger syndrome"	528
S82	AB Dementia	40,825
S83	AB "Parkinson's disease"	13,356
S84	AB "Borderline personality disorder"	2,295
S85	S1 OR S2 OR S3 OR S4 OR S5 OR S6 OR S7 OR S8 OR S9 OR S10 OR S11 OR S12 OR S13 OR S14 OR S15 OR S16 OR S17 OR S18 OR S19 OR S20 OR S21 OR S22 OR S23 OR S24 OR S25 OR S26 OR S27 OR S28 OR S29 OR S30 OR S31 OR S32 OR S33 OR S34 OR S35 OR S36 OR S37 OR S38 OR S39 OR S40 OR S41 OR S42 OR S43 OR S44 OR S45 OR S46 OR S47 OR S48 OR S49 OR S50 OR S51 OR S52 OR S53 OR S54 OR S55 OR S56 OR S57 OR S58 OR S59 OR S60 OR S61 OR S62 OR S63 OR S64 OR S65 OR S66 OR S67 OR S68 OR S69 OR S70 OR S71 OR S72 OR S73 OR S74 OR S75 OR S76 OR S77 OR S78 OR S79 OR S80 OR S81 OR S82 OR S83 OR S84	588,090
S86	MH "Artificial intelligence"	4,563
S87	AB "Artificial intelligence"	2,515
S88	MH "Machine learning"	1,704
S89	AB "machine learning"	4,867
S90	MH "Deep learning"	437
S91	AB "Deep learning"	1,387
S92	MH "Neural network*"	2,446
S93	AB "Neural network*"	3,786
S94	MH "supervised learning"	0
S95	AB "supervised learning"	189
S96	AB "unsupervised learning"	79
S97	MH "Natural language processing"	1,900

S98	AB "Natural language processing"	1,120
S99	S86 OR S87 OR S88 OR S89 OR S90 OR S91 OR S92 OR S93 OR S94 OR S95 OR S96 OR S97 OR S98	16,601
S100	MH "systematic review"	88,705
S101	AB "systematic review"	46,958
S102	AB "systematic literature review"	4,136
S103	MH "meta-analysis"	0
S104	AB "meta-analysis"	43,922
S105	S100 OR S101 OR S102 OR S103 OR S104	133,782
S106	S85 AND S99 AND S105	42

Database: Cochrane Library

ID	Search	Hits
#1	MeSH descriptor: [Artificial Intelligence] explode all trees	1030
#2	("artificial intelligence"):ti,ab,kw	564
#3	MeSH descriptor: [Machine Learning] explode all trees	83
#4	("machine learning"):ti,ab,kw	1066
#5	MeSH descriptor: [Deep Learning] explode all trees	12
#6	("deep learning"):ti,ab,kw	388
#7	MeSH descriptor: [Neural Networks, Computer] explode all trees	118
#8	("neural network"):ti,ab,kw in Cochrane Reviews	0
#9	MeSH descriptor: [Natural Language Processing] explode all trees	9
#10	("natural language processing"):ti,ab,kw	136
#11	("supervised learning"):ti,ab,kw	30
#12	("unsupervised learning"):ti,ab,kw	10
#13	#1 OR #2 OR #3 OR #4 OR #5 OR #6 OR #7 OR #8 OR #9 OR #10 OR #11 OR #12 in Cochrane Reviews	10

Database: Prospero

#	Query	Hits

#1	MeSH DESCRIPTOR Artificial Intelligence	60
#2	"artificial intelligence"	277
#3	MeSH DESCRIPTOR Machine Learning	74
#4	"Machine learning"	372
#5	MeSH DESCRIPTOR Deep Learning	12
#6	"Deep learning"	132
#7	MeSH DESCRIPTOR Neural Networks, Computer	3
#8	MeSH DESCRIPTOR Supervised Machine Learning EXPLODE ALL TREES	1
#9	"supervised learning"	7
#10	MeSH DESCRIPTOR Unsupervised Machine Learning	0
#11	"unsupervised learning"	10
#12	"Natural language processing"	28
#13	#1 OR #2 OR #3 OR #4 OR #5 OR #6 OR #7 OR #8 OR #9 OR #10 OR #11 OR #12	505
#14	* AND (Review_Ongoing OR Review_Completed_not_published OR Review_Completed_published):RS AND (Ccare_of_the_elderly OR Mental health and behavioural conditions OR Violence and abuse):HA NOT Cochrane:DB NOT Animal:DB	1016
#15	#13 AND #14	9

**Database**	**Query**	**Hits**

**APA PsycInfo (EBSCO)**	((MH "mental health") OR (AB "mental health") OR (MH "mental disorder*") OR (AB "mental disorder*") OR (AB "mood disorder*") OR (MH "anxiety disorder*") OR (AB "anxiety disorder*") OR (MH "depressive disorder*") OR (AB "depressive disorder*") OR (MH "Bipolar disorder*") OR (AB "Bipolar disorder*") OR (MH "psychotic disorder*") OR (AB "psychotic disorder*") OR (MH "obsessive-compulsive disorder*") OR (AB "obsessive-compulsive disorder*") OR (MH "panic disorder*") OR (AB "panic disorder*") OR (MH "phobic disorder*") OR (MH "phobic disorder*") OR (MH "substance-related disorder*") OR (AB "substance-related disorder*") OR (MH "Personality disorder*") OR (AB "Personality disorder*") OR (MH "post-traumatic stress disorder*") OR "S25" OR (MH "Attachment disorder*") OR (AB "Attachment disorder*") OR (MH "eating disorder*") OR (AB "eating disorder*") OR (MH "Sleep disorder*") OR (AB "Sleep disorder*") OR (MH "Disruptive Disorder*") OR (AB "Disruptive Disorder*") OR (MH "Adjustment disorder*") OR (AB "Adjustment disorder*") OR (MH "Dissociative disorder*") OR (AB "Dissociative disorder*") OR (MH "Somatic symptom disorder*") OR (AB "Somatic symptom disorder*") OR (MH "somatoform disorder*") OR (AB "somatoform disorder*") OR (MH "Neurocognitive Disorder*") OR (AB "Neurocognitive Disorder*") OR (MH "Autistic Disorder*") OR (AB "Autistic Disorder*") OR (MH "Factitious Disorder*") OR (AB "Factitious Disorder*") OR (MH Delirium) OR (AB Delirium) OR (AB anxious) OR (AB phobi*) OR (MH depression) OR (AB depression) OR (AB melancholia) OR (AB Mania) OR (AB autism) OR (MH "Attention-Deficit Hyperactivity Disorder") OR (AB "Attention-Deficit Hyperactivity Disorder") OR (AB bipolar) OR (AB schizophrenia) OR (AB Stress) OR (AB paranoia) OR (AB psychosis) OR (AB anorexia) OR (AB binge-eating) OR (AB bulimia) OR (AB "drug dependence") OR (AB "substance dependence") OR (AB addiction) OR (AB "drug abuse") OR (AB "substance abuse") OR (AB "Narcolepsy") OR (AB Insomnia) OR (AB Hypersomnolence) OR (AB Parasomnias) OR (AB Kleptomania) OR (AB Pyromania) OR (AB "Intermittent explosive disorder") OR (AB "Oppositional Defiant Disorder") OR (AB "Alzheimer's disease") OR (AB "Asperger syndrome") OR (AB Dementia) OR (AB "Parkinson's disease") OR (AB "Borderline personality disorder")) AND ((MH "Artificial intelligence") OR (AB "Artificial intelligence") OR (MH "Machine learning") OR (AB "machine learning") OR (MH "Deep learning") OR (AB "Deep learning") OR (MH "Neural network*") OR (AB "Neural network*") OR (MH "supervised learning") OR (AB "supervised learning") OR (AB "unsupervised learning") OR (MH "Natural language processing") OR (AB "Natural language processing")) AND ((MH "systematic review")	51

	OR (AB "systematic review") OR (AB "systematic literature review") OR (MH "meta-analysis") OR (AB "meta-analysis"))	
**Scopus**	(TITLE-ABS-KEY ("Artificial intelligence" OR "Machine learning" OR "Deep learning" OR "Neural network*" OR "supervised learning" OR "unsupervised learning" OR "Natural language processing")) AND (TITLE-ABS-KEY ("systematic review" OR "systematic literature review" OR "meta-analysis")) AND ((TITLE-ABS-KEY ("mental health" OR "mental disorder*" OR "mood disorder*" OR "anxiety disorder*" OR "depressive disorder*" OR "Bipolar disorder*" OR "psychotic disorder*" OR "obsessive-compulsive disorder*" OR "panic disorder*" OR "phobic disorder*")) OR (TITLE-ABS-KEY ("substance-related disorder*" OR "Personality disorder*" OR "post-traumatic stress disorder*" OR "Attachment disorder*" OR "eating disorder*" OR "Sleep disorder*" OR "Disruptive Disorder*" OR "Adjustment disorder*" OR "Dissociative disorder*")) OR (TITLE-ABS-KEY ("Somatic symptom disorder*" OR "somatoform disorder*" OR "Neurocognitive Disorder*" OR "Autistic Disorder*" OR "Factitious Disorder*" OR delirium OR anxious OR phobi* OR depression OR melancholia OR mania OR autism)) OR (TITLE-ABS-KEY ("Attention-Deficit Hyperactivity Disorder" OR bipolar OR schizophrenia OR stress OR paranoia OR psychosis OR anorexia OR binge-eating OR bulimia OR "drug dependence" OR "substance dependence" OR addiction OR "drug abuse" OR "substance abuse" OR narcolepsy)) OR (TITLE-ABS-KEY (insomnia OR hypersomnolence OR parasomnias OR kleptomania OR pyromania OR "Intermittent explosive disorder" OR "Oppositional Defiant Disorder" OR "Alzheimer's disease" OR "Asperger syndrome" OR dementia OR "Parkinson's disease")) OR (TITLE-ABS-KEY ("Borderline personality disorder"))) AND (LIMIT-TO (LANGUAGE , "English"))	384
**ACM Library**	[[Abstract: "mental health"] OR [Abstract: "mental disorder*"] OR [Abstract: "mood disorder*"] OR [Abstract: "anxiety disorder*"] OR [Abstract: "depressive disorder*"] OR [Abstract: "bipolar disorder*"] OR [Abstract: "psychotic disorder*"] OR [Abstract: "obsessive-compulsive disorder*"] OR [Abstract: "panic disorder*"] OR [Abstract: "phobic disorder*"] OR [Abstract: "substance-related	1

	disorder*"] OR [Abstract: "personality disorder*"] OR [Abstract: "post-traumatic stress disorder*"] OR [Abstract: "attachment disorder*"] OR [Abstract: "eating disorder*"] OR [Abstract: "sleep disorder*"] OR [Abstract: "disruptive disorder*"] OR [Abstract: "adjustment disorder*"] OR [Abstract: "dissociative disorder*"] OR [Abstract: "somatic symptom disorder*"] OR [Abstract: "somatoform disorder*"] OR [Abstract: "neurocognitive disorder*"] OR [Abstract: "autistic disorder*"] OR [Abstract: "factitious disorder*"] OR [Abstract: delirium] OR [Abstract: anxious] OR [Abstract: phobi*] OR [Abstract: depression] OR [Abstract: melancholia] OR [Abstract: mania] OR [Abstract: autism] OR [Abstract: "attention-deficit hyperactivity disorder"] OR [Abstract: bipolar] OR [Abstract: schizophrenia] OR [Abstract: stress] OR [Abstract: paranoia] OR [Abstract: psychosis] OR [Abstract: anorexia] OR [Abstract: binge-eating] OR [Abstract: bulimia] OR [Abstract: "drug dependence"] OR [Abstract: "substance dependence"] OR [Abstract: addiction] OR [Abstract: "drug abuse"] OR [Abstract: "substance abuse"] OR [Abstract: narcolepsy] OR [Abstract: insomnia] OR [Abstract: hypersomnolence] OR [Abstract: parasomnias] OR [Abstract: kleptomania] OR [Abstract: py]] AND [[Abstract: "artificial intelligence"] OR [Abstract: "machine learning"] OR [Abstract: "deep learning"] OR [Abstract: "neural network*"] OR [Abstract: "supervised learning"] OR [Abstract: "unsupervised learning"] OR [Abstract: "natural language processing"]] AND [[Abstract: "systematic review"] OR [Abstract: "systematic literature review"] OR [Abstract: "meta-analysis"]]	
**IEEE Xplore**	((((Abstract:"mental health" OR "mental disorder*" OR "mood disorder*" OR anxiety OR "Bipolar disorder*" OR "psychotic disorder*" OR "obsessive-compulsive" OR "panic disorders" OR "post-traumatic stress" OR "eating disorders" OR phobia OR depression OR autism OR "Attention-Deficit Hyperactivity" OR bipolar OR schizophrenia OR anorexia OR binge-eating OR bulimia OR Insomnia OR "Alzheimer's" OR Dementia OR "Parkinson's"))) AND ((All Metadata:"systematic review" OR "meta-	6

	analysis"))) AND ((Abstract:"Artificial intelligence" OR "Machine learning" OR "Deep learning" OR "Neural network*"))	
**JBI Evidence Synthesis**	"Artificial intelligence" OR "Machine learning" OR "Deep learning" OR "Neural network*" OR "supervised learning" OR "unsupervised learning" OR "Natural language processing"	0
**Epistemonikos**	(title:("mental health" OR "mental disorder*" OR "mood disorder*" OR "anxiety disorder*" OR "depressive disorder*" OR "Bipolar disorder*" OR "psychotic disorder*" OR "obsessive-compulsive disorder*" OR "panic disorder*" OR "phobic disorder*" OR "substance-related disorder*" OR "Personality disorder*" OR "post-traumatic stress disorder*" OR "Attachment disorder*" OR "eating disorder*" OR "Sleep disorder*" OR "Disruptive Disorder*" OR "Adjustment disorder*" OR "Dissociative disorder*" OR "Somatic symptom disorder*" OR "somatoform disorder*" OR "Neurocognitive Disorder*" OR "Autistic Disorder*" OR "Factitious Disorder*" OR Delirium OR anxious OR phobi* OR depression OR melancholia OR Mania OR autism OR "Attention-Deficit Hyperactivity Disorder" OR bipolar OR schizophrenia OR Stress OR paranoia OR psychosis OR anorexia OR binge-eating OR bulimia OR "drug dependence" OR "substance dependence" OR addiction OR "drug abuse" OR "substance abuse" OR Narcolepsy OR Insomnia OR Hypersomnolence OR Parasomnias OR Kleptomania OR Pyromania OR "Intermittent explosive disorder" OR "Oppositional Defiant Disorder" OR "Alzheimer's disease" OR "Asperger syndrome" OR Dementia OR "Parkinson's disease" OR "Borderline personality disorder") OR abstract:("mental health" OR "mental disorder*" OR "mood disorder*" OR "anxiety disorder*" OR "depressive disorder*" OR "Bipolar disorder*" OR "psychotic disorder*" OR "obsessive-compulsive disorder*" OR "panic disorder*" OR "phobic disorder*" OR "substance-related disorder*" OR "Personality disorder*" OR "post-traumatic stress disorder*" OR "Attachment disorder*" OR "eating disorder*" OR "Sleep disorder*" OR "Disruptive Disorder*" OR "Adjustment disorder*" OR "Dissociative disorder*" OR "Somatic symptom disorder*" OR "somatoform disorder*" OR "Neurocognitive Disorder*" OR "Autistic Disorder*" OR "Factitious Disorder*" OR Delirium OR anxious OR phobi* OR depression OR melancholia OR Mania OR	79

	autism OR "Attention-Deficit Hyperactivity Disorder" OR bipolar OR schizophrenia OR Stress OR paranoia OR psychosis OR anorexia OR binge-eating OR bulimia OR "drug dependence" OR "substance dependence" OR addiction OR "drug abuse" OR "substance abuse" OR Narcolepsy OR Insomnia OR Hypersomnolence OR Parasomnias OR Kleptomania OR Pyromania OR "Intermittent explosive disorder" OR "Oppositional Defiant Disorder" OR "Alzheimer's disease" OR "Asperger syndrome" OR Dementia OR "Parkinson's disease" OR "Borderline personality disorder")) AND (title:("Artificial intelligence" OR "Machine learning" OR "Deep learning" OR "Neural network*" OR "supervised learning" OR "unsupervised learning" OR "Natural language processing") OR abstract:("Artificial intelligence" OR "Machine learning" OR "Deep learning" OR "Neural network*" OR "supervised learning" OR "unsupervised learning" OR "Natural language processing")) AND (title:("systematic review" OR "systematic literature review" OR "meta-analysis") OR abstract:("systematic review" OR "systematic literature review" OR "meta-analysis"))	
**Google Scholar**	("mental health" OR "mental disorder*" OR anxiety OR phobia OR depression OR autism OR "Alzheimer's" OR Dementia OR "Parkinson's") AND ("Artificial intelligence" OR "Machine learning" OR "Deep learning") AND ("systematic review" OR "meta-analysis")	50

Supplementary Table 3: Data extraction form

Concept	Definition
**Study Characteristics**	
Author	The first author of the review
Year of publication	The year in which the review was published
Country of publication	The country where the review was published
Type of publication	The medium in which the review was published (e.g., conference proceedings, peer-reviewed journal, thesis)
Study aim	What the review aimed to find out
Protocol registration	Does the review have a registered protocol
Followed guidelines	What are the guidelines (e.g., PRISMA) that the review follows
**Used methods**	
Target disorders	The target mental disorders that the review focused on
AI approach	The AI approach (i.e., supervised machine learning, unsupervised machine learning, deep learning) that the review focused on
Type of data	Type of data (e.g., neuroimaging data, neuropsychological tests, genetic data) that the review focused on
Language of studies	The language restrictions that the review applied in the search
Time limit	Time limits that the review applied in the search
Databases searched	The electronic databases that were searched in the review
Reference list checking	Type of reference list checking (i.e., forward and/or backward) used for identifying other relevant studies
Number of reviewers in study selection	Number of reviewers who selected the retrieved studies
Number of reviewers in data extraction	Number of reviewers who extracted data from the included studies
Number of reviewers in quality assessment	Number of reviewers who appraised the quality of included studies
Quality appraisal tool	The tool (e.g., QUADAS-2, PROBAST) that was used for appraising the quality of the included studies
Meta-analysis	Was meta-analysis carried out?

**Search results and features of models**	
Number of retrieved studies	Number of all identified studies by searching all databases
Number of included studies	Number of studies that were included in the synthesis
Dataset size	The size of the dataset used for model development and validation
Classification algorithm type	Type of the algorithm (e.g., Support Vector Machine, Random Forest, Naive Bayes, k-Nearest Neighbors) used for diagnosing the mental disorders
Type of validation	Type of validation (i.e., internal or external validation methods) used to validate the model.
**Findings of reviews**	
Accuracy	The reported accuracy of the AI model in diagnosing the mental disorders
Sensitivity	The reported sensitivity of the AI model in diagnosing the mental disorders
Specificity	The reported specificity of the AI model in diagnosing the mental disorders
Area under the curve	The reported area under the curve of the AI model in diagnosing the mental disorders
